# Book Reviews

**Published:** 1905-07

**Authors:** 


### BOOK REVIEWS.

MATERNITAS. A Book Concerning the Care of the Mother and Her Child. By Chas. E. Paddock, M.D., Prof. of Obstetrics, Chicago Post-Graduate Medical School. 186 pages. 5x8. Chicago. C. J. Head & Co., 1905. Price, $1.25.

This little book is intended as a guide to young mothers in the care of the baby and contains much sound advice, which, if followed, will doubtless preserve the future generations, many of whom might otherwise perish through ignorance and mismanagement.

MODERN CLINICAL MEDICINE—INFECTIOUS DISEASES. Edited by J. C. Wilson, A.M., M.D., Philadelphia. An authorized translation from "Die Dentsche Klinik" under the general editorial supervision of Julius L. Salinger, M.D. With two colored plates and sixty illustrations in the text. New York and London, D. Appleton & Co., 1905.

This is the first volume of the modern Clinical Medicine Series to be published by Messrs. Appleton and is devoted to infectious diseases. The matter has been culled from Leyden and Klemperer's Deutsche Klinik. The subjects covered are Typhoid Fever, Klemperer & Liebermeister; Paratyphoid by A. Brion; Typhus and Relapsing Fever, by Lichtheim; Dengue, Vaccinia, Vaccination, Varicella and Weil's Disease, by J. C. Wilson; Yellow Fever and Malta Fever, by Salinger; Influenza, by Furbinger; Influenza since the last pandemic, Ortner. Malarial diseases and Scarlet Fever by Loffler; Measles by Heubner; Smallpox and Acute Articular Rheumatism, by Baumler, Erysipelas by Schutze; Epidemic Cerebro-Spinal Meningetis by Eichhorst; Diphtheria and Pertussis by Baginsky; Pneumonia by Von Leyden; Tuberculosis by Cornet; Lepsis by Purgensen; Dysentery and Ameba Enteritis by Hoppe—Seyler; Cholera Morbus and Cholera Indica by Rumpf; The Plague by Kolle; Mumps by Falkenheim; Angina by Mosse;

Tetanus by P. Jacob; Hydrophobia by Penzoldt; Anthrax, Glanders, Foot and Mouth Disease, Actinomycosis by A. Nicolaier.

The volume contains over nine hundred pages, which allows ample space for a full discussion of the subjects. The authoritative utterances of some of the master minds of medicine have been collected and it is needless to say that the book is a storehouse of information upon one of the most important classes of morbid manifestations—infectious diseases.

---

STIMSON ON FRACTURES AND DISLOCATIONS. A Treatise on Fractures and Dislocations. For Students and Practitioners. By Lewis A. Stimson, B.A., LL.D., Professor of Surgery in Cornell University Medical College, New York; Surgeon to the New York and Hudson Street Hospitals, etc. New (4th) edition, thoroughly revised. Octavo, 844 pages, 331 engravings and 46 full-page plates. Cloth, $5.00, *net;* leather, $6.00, *net;* half morocco, $6.50, *net.* Lea Brothers & Co., Publishers, Philadelphia and New York, 1905.

Since the publication of the third edition many interesting details, some of much practical importance, have been added to the knowledge of certain forms of fracture, particularly in or near joints. The X-rays have been used so freely that a greater degree of confidence may be placed in the accuracy of diagnoses than was formerly possible. In fact, sufficient details have been obtained in some of the rarer forms of injury to permit systematic descriptions. Much new matter of great importance will also be found regarding the operative reduction of old dislocations.

The frequency and severity of the injuries treated in this volume, the necessity for prompt attention, and, finally, their medico-legal possibilities, all unite to render Dr. Stimson's authoritative work essential to general practitioners as well as surgeons. It covers every known form of these lesions, not a few of which were first described in its pages. The author's vast experience and sound judgment are reflected in a literary style of exceptional clearness, and his pages abound in telling engravings and plates. He has endeavored to adapt his work specifically to the needs of the practitioner, particularly in the sections on diagnosis and treatment. In this new and thoroughly revised edition the profession have at command the leading authority upon both subjects in their latest development.

---

WELCH & SCHAMBERG ON ACUTE CONTAGIOUS DISEASES. By William M. Welch, M.D., Consulting Physician to the Municipal Hospital for Contagious and Infectious Diseases, Philadelphia; and Jay F. Schamberg, A.B., M.D., Professor of Dermatology and of Infectious Eruptive Diseases, Philadelphia Polyclinic; Consulting Physician to the Municipal Hospital for Contagious and Infectious Diseases. In one volume of 781 pages, illustrated with 109 engravings and 61 full-page plates. Cloth, $5.00; leather, $6.00, net; half morocco, $6.50 net. Lea Brothers & Co., Philadelphia and New York, 1905.

This book is especially welcome and valuable as an extensive treatise on certain contagious diseases, concerning which extensive text-books are unconvincingly brief. The diseases treated of are smallpox, scarlet fever, diphtheria, typhus fever, chicken-pox, measles and rubella. The authors have had very extensive experience in the treatment of these affections and have given at much length the result of their observations. Pathology and diagnosis, the latter being of such paramount importance in preventive medicine, are very fully discussed. The book is fully illustrated chiefly with original photographs.

---

LEA'S SERIES OF MEDICAL EPITOMES. Edited by Victor C. Pedersen, M.D.

ARNEILL'S EPITOME OF CLINICAL DIAGNOSIS AND URINALYSIS. A Manual for Students and Practitioners. By James R. Arneill, A.B., M.D., Professor of Medicine and Clinical Medicine in the University of Colorado, Physician to the County Hospital and to St. Joseph's Hospital, Denver. In one 12mo volume of 244 pages, with 79 engravings and a colored plate. Cloth, $1.00, net. Lea Brothers & Co., Publishers, Philadelphia and New York, 1905.

Lea's Series of Medical Epitomes will comprise twenty-two volumes, of which this is the seventeenth. The volumes are so uniformly excellent, modern and trustworthy, and so well adapted to the needs of students and preparation for college and State Board examinations that they have practically monopolized the entire "compend" market.

Dr. Arneill has furnished a work that is a fit companion to the others of this series.

It contains an enormous amount of up-to-date information on laboratory investigations and clinical diagnosis, skillfully condensed, simply and clearly stated. As might be expected, the sections on the Blood and Urine are very full, but sufficient consideration is also given to the examination of Stomach Contents, Feces, Sputum, many Bacilli, Cerebro-Spinal Fluid, Milk, etc.

In every line the book is practical, the author never losing sight of the fact that it is intended for the student of medicine and for the practitioner who may not have acquired the ability to put into practice the laboratory methods which so surely lead to precise and correct diagnosis.

An attempt is successfully made to explain fully the most important tests and procedures, and the author very happily anticipates the mistakes and difficulties of the inexperienced worker.

Illustrations are freely used wherever they can help to a better understanding of the text.

---

### THE RATIONAL REDUCTION AND FIXATION OF MAXILLARY FRACTURES.

W. J. Lederer says that the most satisfactory form of apparatus for the fixation of fractures of the lower jaw is the double interdental splint. He describes the technic of taking an impression of the teeth of both jaws in the modeling compound and of producing the finished splint from the molds so obtained. The results secured by this plan of fixation are always good, and the author illustrates the modifications sometimes necessary by describing the details of an unusually difficult case.—*Medical Record, November 19, 1904.*

